# Medically Unexplained Symptoms among Adults from Russia: An Assessment using the Patient Health Questionnaire-15

**DOI:** 10.11621/pir.2023.0203

**Published:** 2023-06-15

**Authors:** Alena A. Zolotareva

**Affiliations:** a School of Psychology, HSE University, Moscow, Russia

**Keywords:** Patient Health Questionnaire-15, factor structure, measurement invariance, psychometric properties, medically unexplained symptoms

## Abstract

**Background:**

The Patient Health Questionnaire-15 (PHQ-15) is one of the most frequently used instruments to measure medically unexplained symptoms in the general population, as well as in groups of patients with mental and physical health problems.

**Objective:**

This study aimed to examine the psychometric properties of the PHQ-15 in assessing a Russian community sample.

**Design:**

A total of 1153 Russian adults age 18 or older participated in this cross-sectional study. They completed the Russian versions of the PHQ-15 and Symptom Check List-90-Revised, SCL-90-R (SCL-90-R). Exploratory and confirmatory factor analyses were used to examine the factor structure of the Russian PHQ-15, and multi-group confirmatory factor analyses were used to test measurement invariance across sex and age. Cronbach’s alpha coefficients and Pearsons Correlation Coefficients were used to evaluate the internal reliability and convergent validity of the Russian PHQ-15.

**Results:**

Exploratory factor analysis revealed a three-factor solution highlighting pain-fatigue, gastrointestinal, and cardiopulmonary symptoms. Confirmatory factor analysis confirmed a bifactor structure for the Russian PHQ-15 merging general and specific somatic symptoms. A multi-group confirmatory factor analysis showed partial invariance across sex and age. The Russian PHQ-15 demonstrated acceptable Cronbach’s alpha coefficients ranging from 0.72 to 0.75 for specific factors and a good Cronbach’s alpha for the total score (a = 0.85), proving the questionnaire’s internal reliability. Finally, positive correlations between the PHQ-15 and SCL-90-R dimensions, and positive intercorrelations between PHQ-15 specific factors, suggested convergent validity.

**Conclusion:**

The Russian PHQ-15 is a reliable and valid instrument for assessing medically unexplained symptoms in the general population. This instrument can be used in diagnostic and counseling settings.

## Introduction

### Medically unexplained symptoms

Medically unexplained symptoms refer to mysterious somatic complaints, the nature of which physicians cannot attribute to any specific diseases or diagnoses. Reid et al. (2003) identified three criteria for a medically unexplained episode: a) the patient has physical symptoms; b) the patient has been medically examined; and c) clinical examination revealed either no abnormality or abnormalities that were thought to be trivial or incidental (p. 520).

These symptoms are an irritant for patients, physicians, and public health systems alike. Epidemiological studies have established that medically unexplained symptoms make up two-thirds of all symptoms reported by people consulting primary care physicians, with the prevalence of somatoform disorders as high as 22.9% for one year, and their comorbidity with at least one other psychiatric disorder in 43.2% of cases (Steinbrecher et al., 2011). Overall, medically unexplained symptoms persisted or worsened in 67% of these primary care patients after one year and in 48.7% after five years (van Westrienen et al., 2019). Although the mean time for recognizing medically unexplained symptoms is 2 to 4 minutes (Houwen et al., 2020), these patients require special attention to their medical history and personal circumstances, advertence to symptoms, and communication with the general practitioner (Houwen et al., 2017). As Rasmussen (2020) noted, these symptoms represent a “junk drawer” in which the general practitioner stores the accumulated data about the patient, which he does not yet know how to categorize or process. Finally, public health systems spend significant resources on the diagnosis and treatment of medically unexplained symptoms (Poloni et al., 2019).

### Measurement of medically unexplained symptoms

In 2013 Zijlema et al. published a systematic review of 40 instruments to measure self-reported medically unexplained symptoms. Based on the criteria of usability and the burden on respondents, they concluded that the Patient Health Questionnaire-15 (PHQ-15) and Symptom Checklist-90 somatization scale are the most suitable for large-scale studies of medically unexplained symptoms. An additional advantage of the PHQ-15 is that it is suitable for evaluating DSM somatic diagnoses (Liao et al., 2016).

The PHQ-15 was developed as a short version of the Primary Care Evaluation of Mental Disorders (PRIME-MD) (Spitzer et al., 1999) for the purpose of evaluating the 15 most common physical complaints in primary care (Kroenke et al., 2002). Later, psychometric studies showed that two items could be excluded from the PHQ-15 due to their gender-specific content (item 4; menstrual problems or other problems with a womans period) and low incidence in the population (item 8; fainting spells) (Witthoft et al., 2013). In addition, several studies confirmed that the PHQ-15 evaluates both general somatization and specific pain-fatigue, gastrointestinal, and cardiopulmonary symptoms (Cano-Garcia et al., 2020; Claassen-van Dessel et al., 2017; Witthoft et al., 2016). This makes the PHQ-15 effective and convenient in assessing medically unexplained symptoms in oncology (Tang et al., 2017), cardiology (Kohlmann et al., 2013), rheumatology (Wolfe et al., 2014), gastroenterology (Derwa et al., 2018), hepatic practice (Sockalingam et al., 2013), pain management (Lanzara et al., 2020), and primary care settings (van Ravesteijn et al., 2009).

The basic psychometric properties of the PHQ-15 were confirmed when the instrument was adapted for Arabic (AlHadi et al., 2017), Chinese (Zhang et al., 2016), Dutch (Terluin et al., 2022), German (Leonhart et al., 2018), Korean (Han et al., 2009), Spanish (Montalban et al., 2010), and Swedish (Nordin et al., 2013) populations. The diagnostic accuracy of the PHQ-15 was proven on samples of participants from the general population (Laferton et al., 2017), outpatients from general hospitals (Cao et al., 2022), and outpatients from a clinic for the treatment of affective, anxiety, eating, and somatoform disorders (Toussaint et al., 2020).

Due to the obvious advantages of the PHQ-15 and the lack of its Russian version, the aim of this study was to adapt the PHQ-15 for a Russian community sample.

## Methods

### Participants

The data were collected by Anketolog, a company that collects empirical data in Russia. The criteria for inclusion in the sample were as follows: 1) 18 years of age or older; 2) native Russian speaker; and 3) residence in Russia during the period of the study. All respondents received a financial reward for participating in the study.

The baseline participant characteristics are displayed in *Table 1*. A total sample of 1153 Russian adults (51.4% females) age 18 to 84 years (M = 41.45, SD= 12.56) participated in this study.

**Table 1:** Baseline characteristics of study participants

Characteristics	n (%)
Sex	
Male, n (%)	560 (48.6)
Female, n (%)	593 (51.4)
Age	
18-30 years	297 (25.8)
31-45 years	551 (47.8)
46-84 years	305 (26.4)
Marital status	
Single, n (%)	303 (26.3)
Married, n (%)	643 (55.8)
Divorced, n (%)	170 (14.7)
Widowed, n (%)	37 (3.2)
Parental status	
No children, n (%)	387 (33.6)
One child, n (%)	382 (33.2)
Two children, n (%)	309 (26.8)
Three children or more, n (%)	73 (6.4)
Education level	
Basic school qualifications, n (%)	93 (8.1)
Vocational training qualifications, n (%)	236 (20.5)
Higher education qualifications, n (%)	802 (69.5)
Doctor degrees, n (%)	22 (1.9)

*Note, n = absolute frequency; % = relative frequency*.

### Instruments

Participants filled out a questionnaire containing a block of socio-demographic questions (sex, age, marital status, parental status, and educational level) and the following instruments:

#### The Patient Health Questionnaire-15 (PHQ-15)

 The PHQ-15 is a 15-item measure assessing medically unexplained symptoms via a list of 15 common physical complaints heard in a primary care setting (Kroenke et al., 2002). These symptoms include 1) stomach pain; 2) back pain; 3) pain in arms, legs, or joints; 4) menstrual cramps or other problems during a womans period; 5) headaches; 6) chest pain; 7) dizziness; 8) fainting spells; 9) heart pounding or racing; 10) shortness of breath; 11) pain or problems during sexual intercourse; 12) constipation, loose bowels, or diarrhea; 13) nausea, gas, or indigestion; 14) feeling tired or having low energy; and 15) trouble sleeping. Each somatic symptom is rated on a three-point Likert scale which ranges from 0 (“not bothered at all”) to 2 (“bothered a lot”). Based on a recent study highlighting the shortcomings of back translation (Behr, 2017), the original version of the PHQ-15 was translated into Russian by two Russian-speaking specialists in psychosomatic medicine.

#### The Symptom Check List-90-Revised (SCL-90-R)

 The SCL-90-R is a 90-item measure assessing nine dimensions of psychological distress: 1) somatization (headaches, chest pain, nausea, etc.); 2) obsessive-compulsive (obsessive thoughts and uncontrolled behavior); 3) interpersonal sensitivity (feeling of personal inadequacy and inferiority); 4) depression (dysphoria, suicidal ideas and intentions); 5) anxiety (nervousness, tension, panic attacks); 6) hostility (aggression, irritability, etc.); 7) phobic anxiety (fear of a particular person, place or object); 8) paranoid ideation (delirium, suspicion, etc.); and 9) psychoticism (symptoms ranging from schizoid to clinical psychosis). The SCL-90-R assesses three summary outcomes it identifies as global scores: the Global Severity Index (GSI), the Positive Symptom Distress Index (PSDI), and the Positive Symptom Total (PST) (Derogatis, 1994). Each item describes a symptom that is rated on a five-point Likert scale which ranges from 1 (“not at all”) to 5 (“extremely”). Tarabrina (2001) assessed the psychometric properties of the SCL-90-R in Russian clinical and non-clinical samples, including students, emigrants, war veterans, bank employees, patients with schizophrenia, and patients with somatoform disorders.

### Data analysis

The data were analyzed in six steps using the AMOS and SPSS version 27.0. First, preliminary analyses calculated the frequencies and percentages for categorical variables and the means and standard deviations for the numerical variables. Second, exploratory factor analysis (EFA) was used to examine the factor structure of the Russian PHQ-15. Specific tests such as Kaiser-Meyer-Olkin (KMO) test for measure of sampling adequacy and Chi square for Bartletts test of sphericity were used to evaluate the data’s suitability. In particular, KMO values greater than 0.50 and significant Chi squares for Bartlett’s test of sphericity were considered suitable for EFA (Tabachnick & Fidell, 2007).

Third, confirmatory factor analysis (CFA) was used to test different factor solutions for the Russian PHQ-15. The goodness of fit of the CFA models was measured via three fit indexes: the comparative fit index (CFI), the Tucker Lewis index (TLI), and the root mean square error of approximation (RMSEA). A model was considered fit when CFI and TLI values were greater than 0.90, and RMSEA values were less than 0.80 (Hu & Bentler, 1999).

Fourth, multi-group CFAs were performed to evaluate the measurement invariance of the Russian PHQ-15. Traditionally, there are three invariance models (Chakraborty, 2017; Milfont & Fischer, 2010): a) the configural invariance model, which shows that respondents from different group conceptualize a phenomenon in the same way; b) the metric invariance model, which assesses whether comparable groups respond to the items in the same way; and c) the scalar invariance model, which compares latent means across different groups. Thus, ACFI between the previous and subsequent models must be equal or below 0.010 (Cheung & Rensvold, 2002).

Fifth, Cronbach’s alpha coefficient was used to assess the internal reliability of the Russian PHQ-15. Values of 0.70 or higher and 0.90 or higher indicate acceptable and excellent internal consistency, respectively (Kline, 1999). Finally, Pearsons correlation coefficient was used to evaluate the convergent validity of the Russian PHQ-15. Regarding magnitude of effect sizes, correlation coefficients greater than 0.10 are small, those of 0.30 are medium, and those of 0.50 are large (Cohen, 1988).

## Results

### Preliminary analyses

The characteristics making up the Russian PHQ-15 items are shown in *Table 2*. Prior to the analysis, item 4 (menstrual cramps or other problems associated with a womans period) was excluded due to its gender-specific content (Kroenke et al., 1998). After the frequency analysis, item 8 (fainting spells) and item 11 (pain or problems during sexual intercourse) were also excluded as rare in this population.

**Table 2:** Characteristics of the Russian PHQ-15 items

	Item	Mean	SD	Cronbach’s α
PHQ01	Stomach pain	0.44	0.57	0.83
PHQ02	Back pain	0.77	0.69	0.84
PHQO3	Pain in arms, legs, or joints	0.72	0.68	0.84
PHQO5	Headaches	0.73	0.64	0.84
PHQ06	Chest pain	0.28	0.51	0.84
PHQ07	Dizziness	0.35	0.56	0.83
PHQ09	Heart pounding or racing	0.40	0.58	0.83
PHQ1O	Shortness of breath	0.33	0.55	0.83
PHQ12	Constipation, loose bowels, or diarrhea	0.32	0.56	0.84
PHQ13	Nausea, gas, or indigestion	0.39	0.59	0.83
PHQ14	Feeling tired or having low energy	0.91	0.71	0.82
PHQ15	Trouble sleeping	0.68	0.72	0.83

*Note. SD = standard deviation; Cronbach a = Cronbach's alpha coefficients if item dropped*.

The frequency of the somatic symptoms is presented in *Table 3*. The PHQ-15 total score (a = 0.85) had good internal consistency, which did not improve when specific items were excluded (with values ranging from 0.82 to 0.84).

**Table 3:** Frequency of somatic symptoms among Russian adults

	Item	n (%)
PHQ01	Stomach pain	456 (39.5)
PHQ02	Back pain	708 (61.4)
PHQ03	Pain in arms, legs, or joints	673 (58.4)
PHQ04	Menstrual problems or other problems with period	283 (24.5)
PHQ05	Headaches	717(62.2)
PHQ06	Chest pain	289 (25.1)
PHQ07	Dizziness	356 (30.9)
PHQ08	Fainting spells	35 (3.0)
PHQ09	Heart pounding or racing	404 (35.0)
PHQ10	Shortness of breath	338 (29.3)
PHQ11	Pain or problems during sexual intercourse	78 (6.8)
PHQ12	Constipation, loose bowels, or diarrhea	313(27.1)
PHQ13	Nausea, gas, or indigestion	391 (33.9)
PHQ14	Feeling tired or having low energy	807 (70.0)
PHQ15	Trouble sleeping	618 (53.6)

*Note: n = absolute frequency; % = relative frequency*.

### Factor structure and measurement invariance

The first step was to disclose the factor structure of the Russian PHQ-15. The results of the EFA suggested that three factors explained over 55% of the variance. The Kaiser-Meyer-Olkin (KMO) test for measure of sampling adequacy showed 0.890, and Chi square for Bartlett’s test of sphericity was significant (χ^2^ = 3775.130, df = 66, p < 0.001). The first factor included item 2 (back pain; λ = 0.78), item 3 (pain in arms, legs, or joints; λ = 0.68), item 5 (headaches; λ = 0.48), item 14 (feeling tired or having low energy; λ = 0.64), and item 15 (trouble sleeping; λ = 0.55). This factor reflected the pain-fatigue symptoms.

The second factor included item 1 (stomach pain; λ = 0.69), item 12 (constipation, loose bowel, or diarrhea; λ = 0.83), and item 13 (nausea, gas, or indigestion; λ = 0.80). This factor expressed the gastro-intestinal symptoms.

The third factor included item 6 (chest pain; λ = 0.73), item 7 (dizziness; λ = 0.50), item 9 (heart pounding or racing; λ = 0.76), and item 10 (shortness of breath; λ = 0.71). This factor represented the cardiopulmonary symptoms.

**Figure 1.**
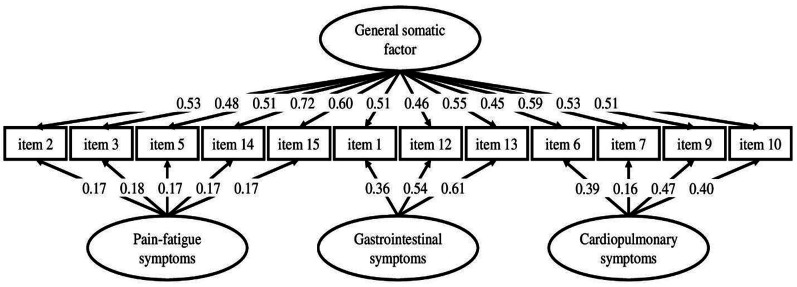
Factor structure of the Russian PHQ-15

The next step was to examine the single-factor, three-factor, and bifactor structure of the Russian PHQ-15. As shown in *Table 2,* the bifactor model had the best fit indexes. Factor loadings for the bifactor structure of the Russian PHQ-15 are displayed in *Figure 1*.

The final step was to assess the measurement invariance with regard to sex and age *(Table 4)*. Regarding sex, the configural invariance, metric invariance, and scalar invariance models fit the data well. However, the ACFI between metric and scalar invariance models were greater than 0.010. Regarding age, the configural invariance, metric invariance, and scalar invariance models also fit the data well. All ACFI were greater than 0.010.

**Table 4:** Factor structure of the Russian PHQ-15 and measurement invariance across sex and age

	χ^2^	df	RMSEA (90% CI)	CFI	TLI	ACFI
Single group solutions						
Model 1. Single-factor structure	626.745*	54	0.096 (0.089-0.102)	0.846	0.812	
Model 2. Three-factor structure	195.679*	51	0.050 (0.042-0.057)	0.961	0.950	
Model 3. Bifactor structure	146.839*	46	0.044 (0.036-0.052)	0.973	0.961	
Invariance models across sex						
Model 4. Configural invariance Model 5. Metric invariance	195.289* 214.663*	93 no	0.031 (0.025-0.037) 0.029 (0.023-0.034)	0.972 0.971	0.960 0.965	0.001
Model 6. Scalar invariance	328.208*	122	0.038 (0.033-0.043)	0.943	0.939	0.028
Invariance models across age						
Model 7. Configural invariance	262.312*	140	0.028 (0.022-0.033)	0.965	0.951	
Model 8. Metric invariance	346.893*	174	0.029 (0.025-0.034)	0.951	0.944	0.014
Model 9. Scalar invariance	527.540*	198	0.038 (0.034-0.042)	0.907	0.907	0.044

*Note. < 0.001. ACFI refers to the change from the configural to the metric models as well as from the metric to the scalar models*.

### Reliability and validity

As presented in *Table 5,* the Russian PHQ-15 subscales significantly correlated with each other and the general somatization index. The intercorrelation values ranged from 0.45 to 0.58, and the correlation values with the total index ranged from 0.74 to 0.90. Cronbach's alpha coefficients were 0.73,0.75, and 0.72 for pain-fatigue, gastrointestinal, and cardiopulmonary symptoms, respectively. As mentioned earlier, the Cronbach's alpha coefficient was 0.85 for the general somatization index.

**Table 5:** Intercorrelations and Cronbach's alpha coefficients for PHQ-15 subscales

PHQ-15 subscales	Pain-symptoms fatigue	Gastroint. symptoms	Cardiopul. symptoms	Cronbach α
Pain-fatigue symptoms				0.73
Gastrointestinal symptoms	0.50*			0.75
Cardiopulmonary symptoms	0.58*	0.45*		0.72
General symptoms	0.90*	0.74*	0.81*	0.85

*Note. < 0.001. Gastroint. = gastrointestinal; Cardiopul. = cardiopulmonary*.

The Russian PHQ-15 scores were also correlated with the SCL-90-R scores *(Table 6)*. All indexes of psychopathology were positively correlated with pain-fatigue (with values ranging from 0.41 to 0.73), gastrointestinal (with values ranging from 0.33 to 0.55), and cardiopulmonary symptoms (with values ranging from 0.43 to 0.73), as well as the general somatization index (with values ranging from 0.51 to 0.82).

**Table 6:** Correlations between the PHQ-15 and SCL-90-R

	Pain-fatigue symptoms	Gastroint. symptoms	Cardiopul. symptoms	General symptoms
Somatization (SOM)	0.73*	0.55*	0.73*	0.82*
Obsessive-compulsive (OBS)	0.59*	0.44*	0.53*	0.64*
Interpersonal sensitivity (INT)	0.55*	0.41*	0.48*	0.59*
Depression (DEP)	0.62*	0.44*	0.54*	0.66*
Anxiety (ANX)	0.58*	0.47*	0.63*	0.68*
Hostility (HOS)	0.52*	0.42*	0.48*	0.58*
Phobic anxiety (PHOB)	0.41*	0.36*	0.50*	0.51*
Paranoid ideation (PAR)	0.47*	0.33*	0.44*	0.51*
Psychoticism (PSY)	0.46*	0.34*	0.47*	0.52*
Global Severity Index (GSI)	0.65*	0.49*	0.63*	0.72*
Positive Symptom Distress Index (PSDI)	0.66*	0.50*	0.60*	0.72*
Positive Symptoms Total (PST)	0.55*	0.35*	0.43*	0.55*

Note. *p < 0.001. Gastroint. = gastrointestinal; Cardiopul. = cardiopulmonary.

## Discussion

The current study aimed to adapt the PHQ-15 for a Russian community sample. Thus, the findings relate to the psychometric properties of the Russian PHQ-15. Preliminary analysis showed that item 4 (menstrual cramps or other problems associated with a womans period), item 8 (fainting spells), and item 11 (pain or problems during sexual intercourse) should be excluded from the questionnaire due to their gender specificity or low frequency in a Russian population. Many previous studies confirmed that items 4 and 8 should be deleted for greater psychometric coherence of the PHQ-15 (Cano-Garda et al., 2020; Leonhart et al., 2018; Witthoft et al., 2013), but only one study suggested that item 11 should also be excluded (Kroenke et al., 1998).

Sexual dysfunctions are fairly common complaints in both clinical and general population settings: 3%-18% people suffered from dyspareunia (Schultz et al., 2005); 10-28% from vulvodynia (Harlow et al., 2014); and 3-76.5% from erectile dysfunction (Kessler et al., 2019). The prevalence of these complaints is up to 51.2-92% among patients with mental and physical diseases (Abdelatti et al., 2020; Dastoorpoor et al., 2021). However, there are strong differences between objective and self-report measures of sexual dysfunctions. People tend to downplay the frequency and significance of their sexual complaints due to misperceptions, lack of knowledge, and personal factors (Takeuchi et al., 2021).

Thus, the final version of the Russian PHQ-15 consisted of 12 items. Factor analyses revealed a bifactor solution with a general somatic symptoms factor and specific factors: pain-fatigue, gastrointestinal, and cardiopulmonary symptoms. Previous psychometric studies demonstrated that the PHQ-15 includes a general somatic burden factor, but with variations of at least seven specific factors: they are neurological, gastrointestinal, and cardiopulmonary symptoms; somatization; pain; fatigue; and pain-fatigue symptoms (Lee et al., 2011; Walentynowicz et al., 2018; Zhang et al., 2016).

The current study also revealed only partial invariance across sex and age, which is consistent with earlier findings (Cano-Garda et al., 2020; Zhou et al., 2020). The possible reason for this could be the well-established fact that there are sex- and age-specific manifestations of the psychosomatic burden. Thus, females reported ‘more intense, more numerous, and more frequent” somatic symptoms than males due to greater visceral sensitivity; special bodily symptom labelling, description, and reporting; and social and cultural circumstances (Barsky et al., 2001, p. 266). A recent study described changing trajectories of functional somatic symptoms from adolescence to middle age, highlighting the close relationship between ageing and the psychosomatic burden (Nummi et al., 2017).

The Russian version of PHQ-15 showed good internal reliability and convergent validity. The evidence was provided by the acceptable Cronbachs alpha coefficients, positive intercorrelations between PHQ-15 specific factors, and positive correlations between the PHQ-15 and SCL-90-R scores, which are considered the two most suitable instruments for assessing medically unexplained symptoms (Zijlema et al., 2013).

## Conclusion

In conclusion, this study revealed that the Russian PHQ-15 is a psychometrically sound instrument for assessing medically unexplained symptoms in a Russian community sample.

## Limitations

This study has a number of limitations. Primarily, its reliance on self-reporting carries the risk of the results being distorted by social desirability, because persons with a high somatic burden tend to view lying as more acceptable (Butean et al., 2020). In addition, this study provided evidence of convergent validity, but predictive validity is more important for a clinical self-reported instrument. An objective criterion for the usability and effectiveness of the PHQ-15 may be its ability to diagnose somatic symptoms and related disorders in accordance with the Diagnostic and Statistical Manual of Mental Disorders (DSM-5) (Toussaint et al., 2020).

Another limitation is that only the SCL-90-R was used to establish the convergent validity of the Russian PHQ-15, whereas other adapted versions have been validated using measures of subjective well-being, self-reported physical health, and health-related quality of life (Kocalevent et al., 2013; Stauder et al., 2021; Wilkie et al., 2018). Finally, this study was population-based, although the PHQ-15 was developed to assess medically unexplained symptoms presented in primary care facilities (Kroenke et al., 1998). One avenue for future research is to test the sensitivity and specificity of the Russian PHQ-15 in clinical settings.
